# A 3D-Printed Polycaprolactone/Marine Collagen Scaffold Reinforced with Carbonated Hydroxyapatite from Fish Bones for Bone Regeneration

**DOI:** 10.3390/md20060344

**Published:** 2022-05-25

**Authors:** Se-Chang Kim, Seong-Yeong Heo, Gun-Woo Oh, Myunggi Yi, Won-Kyo Jung

**Affiliations:** 1 Major of Biomedical Engineering, Division of Smart Healthcare, College of Information Technology and Convergence and New-Senior Healthcare Innovation Center (BK21 Plus), Pukyong National University, Busan 48531, Korea; tpckd181q@pukyong.ac.kr (S.-C.K.); myunggi@pknu.ac.kr (M.Y.); 2 Marine Integrated Biomedical Technology Center, The National Key Research Institutes in Universities, Pukyong National University, Busan 48513, Korea; 3 Jeju Marine Research Center, Korea Institute of Ocean Science & Technology (KIOST), Jeju 63349, Korea; syheo@kiost.ac.kr; 4 National Marine Biodiversity Institute of Korea (MABIK), Seochun, Chungcheongnam 33662, Korea; ogwchobo@mabik.re.kr; 5 Research Center for Marine Integrated Bionics Technology, Pukyong National University, Busan 48513, Korea

**Keywords:** marine collagen, carbonated hydroxyapatite, fishery by-product, 3D scaffold, bone regeneration

## Abstract

In bone tissue regeneration, extracellular matrix (ECM) and bioceramics are important factors, because of their osteogenic potential and cell–matrix interactions. Surface modifications with hydrophilic material including proteins show significant potential in tissue engineering applications, because scaffolds are generally fabricated using synthetic polymers and bioceramics. In the present study, carbonated hydroxyapatite (CHA) and marine atelocollagen (MC) were extracted from the bones and skins, respectively, of *Paralichthys olivaceus*. The extracted CHA was characterized using Fourier transform infrared (FTIR) spectroscopy and X-ray diffraction (XRD) analysis, while MC was characterized using FTIR spectroscopy and sodium dodecyl sulfate-polyacrylamide gel electrophoresis (SDS-PAGE). The scaffolds consisting of polycaprolactone (PCL), and different compositions of CHA (2.5%, 5%, and 10%) were fabricated using a three-axis plotting system and coated with 2% MC. Then, the MC3T3-E1 cells were seeded on the scaffolds to evaluate the osteogenic differentiation in vitro, and in vivo calvarial implantation of the scaffolds was performed to study bone tissue regeneration. The results of mineralization confirmed that the MC/PCL, 2.5% CHA/MC/PCL, 5% CHA/MC/PCL, and 10% CHA/MC/PCL scaffolds increased osteogenic differentiation by 302%, 858%, 970%, and 1044%, respectively, compared with pure PCL scaffolds. Consequently, these results suggest that CHA and MC obtained from byproducts of *P. olivaceus* are superior alternatives for land animal-derived substances.

## 1. Introduction

Bone is a dense, complex, hierarchically structured connective tissue composed of calcified matrix, which includes 65% inorganic materials, 25% organic materials, and 10% water, cells (osteoblasts, osteoclasts, and osteocytes), and various proteins, such as osteocalcin, osteopontin, and osteoprotegerin [1,2]. Bone is one of the most important tissues in the human body and provides mechanical strength, protects vital body organs, and stores and releases minerals; also, bone is continuously subjected to various defects, injuries, and diseases [3]. Some bone-related issues cannot be self-repaired and require special treatments, such as bone grafts including autografts, allografts, and tissue-engineered bone substitutes that promote bone tissue regeneration [4]. Bioceramics including bioactive glass, hydroxyapatite (HA), and calcium phosphate are promising bioactive materials that were successfully used for bone tissue regeneration applications [5].

HA (Ca_10_(PO_4_)_6_(OH)_2_) is the main component of bone and accounts for approximately 60–70% of total bone mass [6,7]. Hence, HA was reported to be a potential therapeutic material and was applied in spinal fusion surgery, bone defect treatment, bone-related surgery, and bone mass augmentation [8,9]. Although HA is a bioactive material that induces bone regeneration, this material has a lack of mechanical stability, high brittleness, and low interaction with cells. Thus, many studies are currently being conducted to composite HA with various synthetic and natural materials to improve its mechanical properties and cellular functions such as cell adhesion, migration, and differentiation [10,11].

Collagen is the main protein in the extracellular matrix (ECM), accounting for approximately one-quarter of total body protein [12,13]. Collagen is a structural protein with a triple-helical structure that is composed of repeated G-X-Y peptide units. There are 29 types of collagen, and the majority are type I, II, and III. Collagen is a particularly promising biomaterial that promotes cell adhesion, proliferation, and differentiation by providing an ECM-mimicking environment [14,15]. However, collagen isolated from land animals, such as pigs and cows, has various barriers to application in medical products, due to religious reasons and zooanthroponoses. Marine-derived collagen is a highly potent alternative to land animal-derived collagen, since there are no religious barriers or reported zooanthroponoses [16,17,18]. In addition, marine-derived collagen has shown a lower immune response, higher water solubility, and lower production costs than land animal-derived collagen [19,20]. Among marine organisms, *Paralichthys olivaceus* may be a good substitute for land animal-derived collagen, because of its good accessibility and availability as a byproduct in the seafood industry in the Republic of Korea [21]. However, investigations of bone regenerative scaffolds fabricated with *P. olivaceus*-derived biomaterials were rarely reported. In addition, unlike jellyfish containing a large amount of collagen type 2 and a marine sponge containing a large amount of collagen type 4, fish skins contain a large amount of collagen type 1, so it can be used as a raw material for biomaterials for tissue regeneration [22]. 

Polycaprolactone (PCL) is a biocompatible, biodegradable synthetic polymer that was approved by the United States Food and Drug Administration (FDA) and is widely used in the fabrication of tissue-engineered substitutes for bone regeneration applications [23,24]. Moreover, PCL has good mechanical properties such as high stiffness and strength, and slow biodegradation time (2–4 years); these properties play key roles in maintaining the appropriate mechanical strength in tissue-engineered bone substitutes fabricated with natural polymers and bioceramics [25,26,27].

A three-axis plotted scaffold provides a three-dimensional (3D) structural environment to facilitate osteocyte adhesion, migration, and differentiation; these scaffolds are subjects of bone regeneration research [28,29]. In particular, 3D printing is an immerging technology that can be applied to fabricate complex and personalized structures; this technique is highly reproducible, compared with other techniques such as gas foaming, hydrogel usage, and electrospinning [30]. Moreover, organic substances, such as alginate, chitosan, gelatin, and collagen, and inorganic substances, such as HA, tricalcium phosphate, and whitlockite are widely used to fabricate bone regenerative scaffolds using 3D printing technology [5,31,32]. Therefore, 3D-printed scaffolds offer benefits for patient treatment through personalization.

In this study, we fabricated a composite 3D-printed scaffold using PCL and carbonated HA (CHA), and enhanced the biological properties using a coating of marine atelocollagen (MC). MC and CHA were extracted from the skin and bones, respectively, which are byproducts of *P. olivaceus*. Sodium dodecyl sulfate-polyacrylamide gel electrophoresis (SDS-PAGE), Fourier transform infrared (FTIR) spectroscopy, and amino acid composition analyses were used to evaluate the characteristics of MC, and FTIR spectroscopy, X-ray diffraction (XRD) analysis, and energy dispersive spectroscopy (EDS) were performed to evaluate the characteristics of CHA. The fabricated CHA/MC/PCL scaffolds were then analyzed to determine their potential for facilitating osteogenic differentiation and bone tissue regeneration through in vitro and in vivo investigations. 

## 2. Results

### 2.1. Extraction and Characterization of MC

**Figure 1:**
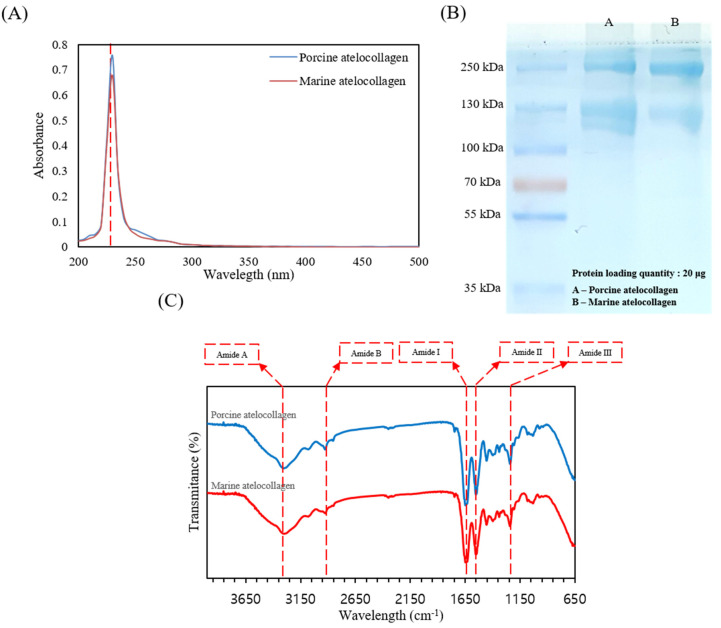
Characterization of collagen extracted from skins of *P. olivaceus* and of porcine commercial atelocollagen. (**A**) UV-Vis spectra of the two types of collagen; (**B**) Sodium dodecyl sulfate-polyacrylamide electrophoresis evidences the molecular structure and organization of two collagen; (**C**) Fourier transform infrared spectra of collagens exhibits the main vibrations of collagen molecular organization.

One simple method to identify collagen is to scan collagen samples on a UV spectrum (200–400 nm), because the triple helical structure of collagen has absorption at 230 nm. As shown in Figure 1A, commercial atelocollagen and MC have a maximum absorption peak at 230 nm, which is associated with COO^−^, CONH_2_, and C=O groups in the polypeptides of collagen. Collagen has a few tryptophan, histidine, tyrosine, and phenylalanine components that absorb UV at 250 nm and 280 nm.

Figure 1B represents the SDS-PAGE components of commercial atelocollagen and MC. Commercial atelocollagen and MC both consist of α_1_ and α_2_ chains and high molecular weight β and γ components. Thus, the extracted commercial atelocollagen and MC correspond to type I collagen. 

The FTIR spectra of commercial atelocollagen and MC from the skins of *P. olivaceus*, which can be used to identify information on the secondary structure of collagen, are shown in Figure 1C. The changes in the spectral peaks were shown to include changes in the amide A and B bands as well as the amide I–III regions. The amide A band at 3300 cm^−1^ and the amide B band peaks at 2972 cm^−1^ are associated with N–H stretching vibrations and the asymmetrical stretching of CH_2_. In addition, the amide I peak at 1635 cm^−1^ is mainly associated with the stretching vibrations of C=O, along with the polypeptide backbone or COO^−^. The amide II region of collagen type I appears at approximately 1549 cm^−1^ from N–H bending vibration coupled with C=N stretching vibrations. The amide III region at 1239 cm^−1^ represents the peaks between C=N stretching vibrations and N–H deformation from amide linkages of CH_2_ groups of the glycine backbone and proline side-chain. As shown in Figure 1C, the intensity and positions of the amide A, B, and I–III bands are similar for the commercial atelocollagen and MC, as well as type I collagen.

### 2.2. Amino Acid Components

Table 1 shows the amino acid components of commercial atelocollagen and MC from *P. olivaceus*. Commercial atelocollagen and MC were found to have glycine (238.3 and 247.3 per 1000, respectively) and to be low in tyrosine, histidine, methionine, and cysteine. The imino acid content of the extracted collagen was 264.3 and 208.6 per 1000 for porcine atelocollagen and marine atelocollagen, respectively. Based on these differences, the mechanical properties of MC are weaker than commercial collagen. Collagen with a greater imino acid content is more stable in the helix structure, due to the contents of proline and hydroxyproline.

### 2.3. Extraction and Characterization of CHA

**Figure 2:**
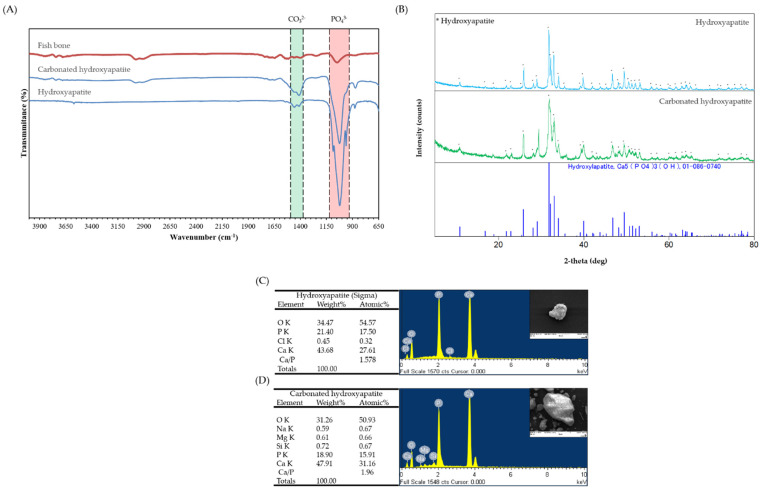
Characterization of hydroxyapatite isolated from frame of *Paralichthys olivaceus* and sigma. (**A**) Fourier transform infrared spectra of HA and CHA; (**B**) X-ray diffraction spectra of HA and CHA. The energy dispersive spectrometer of (**C**) HA and (**D**) CHA.

The FTIR spectra of raw fishbones and HA and CHA from the bones of *P. olivaceus* are shown in Figure 2A. The FTIR bands of raw fishbones were observed at 1047 cm^−1^, 1644–1740 cm^−1^, 2911 cm^−1^, and 2977 cm^−1^; these bands are both minerals and organic compound of fishbones. HA has bands at 878 cm^−1^, 1000–1100 cm^−1^, 1400–1500 cm^−1^, 3447 cm^−1^, and 3571 cm^−1^. The strongest band from 1000–1100 cm^−1^ is the stretching of PO_4_^3−^ vibrations. The band at approximately 1400–1500 cm^−1^ corresponds to the carbonate group of HA and CHA. The band of OH stretching of HA appears from 1000–1100 cm^−1^. CHA also shows all of the bands of PO_4_^3−^, CO_3_^2−^, and OH, and because CHA was extracted by alkaline lysis, the band of CO_3_^2−^ for CHA is more prominent than that for HA (Figure 2A).

XRD analysis is a reliable method for investigating the phase purity and crystallinity of a compound and determining the quantitative and qualitative aspects of a solid compound. Results of XRD are mainly evaluated through comparison with the International Center for Diffraction Data (ICDD) standards. The crystallinity and purity of HA and CHA were defined by XRD analysis. The XRD peaks of the standard and the HA and CHA from the bones of *P. olivaceus* are shown in Figure 2B. The obtained peaks of HA and CHA at 2-theta were identical to 01-086-0740 from the ICDD. The peaks of HA and CHA matched with those of the standard ICDD 01-086-0740 (Hydroxyapatite; Ca_5_(PO_4_)_3_OH) (Figure 2B).

### 2.4. Energy Dispersive Spectrometer (EDS)

Figure 2C,D displays the EDS data that confirm the content of C, O, Na, Mg, Cl, P, and Ca in the powder. The ratio of Ca/P for the HA powder was 1.578, and that for the CHA powder was 1.96. These results show that CHA has carbonate groups, because it was extracted by alkaline hydrolysis. 

### 2.5. Characterization of the CHA-Reinforced Scaffolds

**Figure 3:**
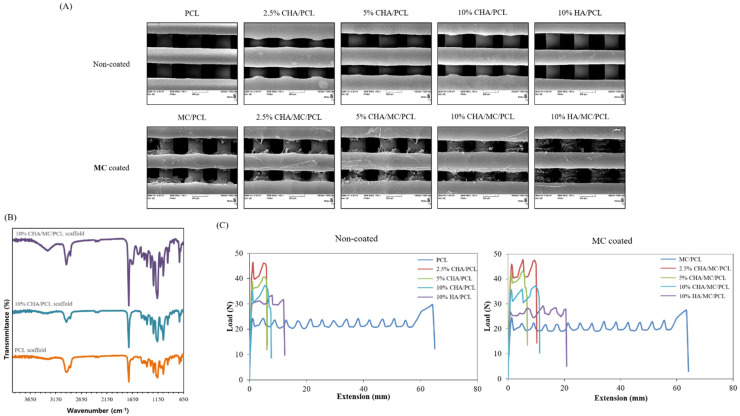
Morphology and characterization of the CHA-reinforced scaffolds. (**A**) SEM image of non-coated group and MC coated group; (**B**) FTIR analysis of non-coated group and MC coated group; (**C**) load-extension curve of non-coated group and MC coated group.

The mean strut diameter of the CHA-reinforced scaffolds was controlled by adjusting the extruding temperature, nozzle diameter, and speed of the extruder. Based on SEM observations, the strut diameters of PCL, 2.5% CHA/PCL, 5% CHA/PCL, 10% CHA/PCL, 10% HA/PCL, MC/PCL, 2.5% CHA/MC/PCL, 5% CHA/MC/PCL, 10% CHA/MC/PCL, and 10% HA/MC/PCL scaffolds were 518.54 ± 5.72 μm, 607.27 ± 3.34 μm, 571.37 ± 16.43 μm, 561.03 ± 16.5 μm, 484.57 ± 19.68 μm, 508.17 ± 11.62 μm, 599.42 ± 2.94 μm, 526.28 ± 15.34 μm, 539.12 ± 15.34 μm, and 466.67 ± 16.04 μm, respectively. For these results, the strut diameter and pore size of the CHA-reinforced scaffolds were fabricated under the same conditions, but slight differences were observed between the scaffolds (Figure 3A, Table 2).

The FTIR spectra of the CHA-reinforced scaffolds were in the spectral range of 4000–650 cm^−1^. The FTIR spectrum of the PCL scaffold was observed at 2860 cm^−1^ (C-H) and 1720 cm^−1^ (C=O) stretching peaks, and the FTIR spectrum of the 10% CHA/PCL scaffold was observed at the same peaks. For the 10% CHA/MC/PCL scaffold, in addition to the peaks of the PCL scaffold, the amide peaks (amide A, B, I–III) of MC were also identified (Figure 3B).

The mechanical properties of the CHA-reinforced scaffolds were evaluated using tensile mechanical testing in a universal testing machine. As shown in Figure 3C, the elastic modulus values of PCL, 2.5% CHA/PCL, 5% CHA/PCL, 10% CHA/PCL, 10% HA/PCL, MC/PCL, 2.5% CHA/MC/PCL, 5% CHA/MC/PCL, 10% CHA/MC/PCL, and 10% HA/MC/PCL scaffolds were 6.29 ± 0.28 MPa, 10.19 ± 0.01 MPa, 9.26 ± 0.33 MPa, 6.85 ± 0.55 MPa, 7.76 ± 0.37 MPa, 6.37 ± 0.16 MPa, 9.38 ± 0.45 MPa, 9.1 ± 0.12 MPa, 7.08 ± 0.52 MPa, and 7.77 ± 0.42 MPa, respectively. The elastic modulus value tended to increase in the scaffolds containing CHA and HA, compared with that of PCL; furthermore, the elastic modulus value decreased as the content of CHA and HA increased. These results may be due to the diameter of the strut and the size of CHA and HA (Figure 3C, Table 2). 

### 2.6. Cell Viability on the CHA-Reinforced Scaffolds

**Figure 4:**
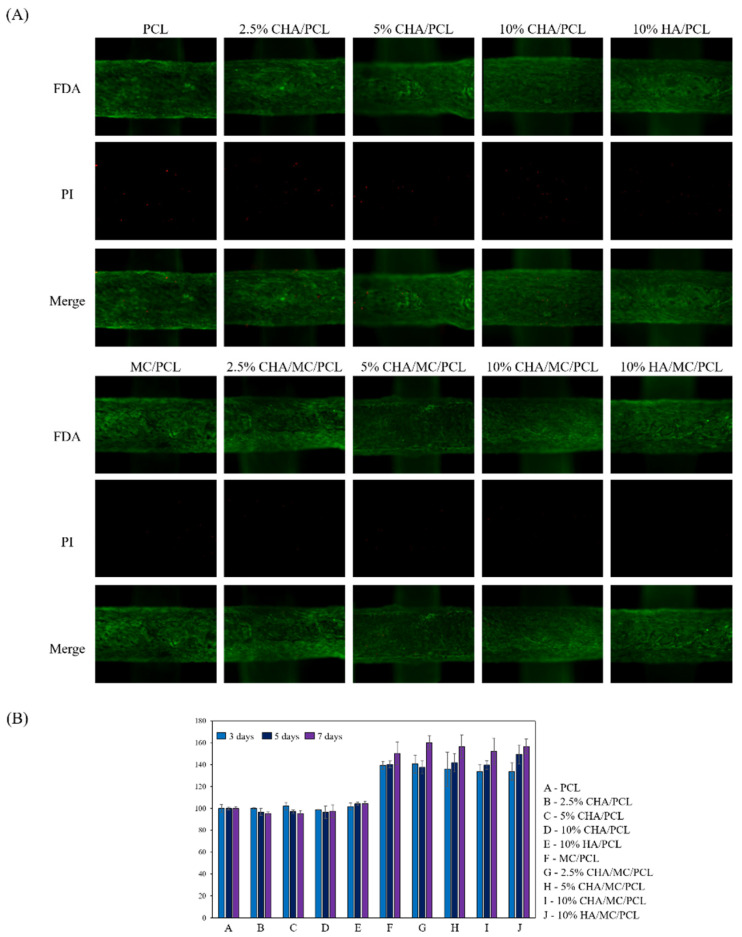
Cell viability of non-coated and MC-coated scaffolds on MC3T3-E1. (**A**) cell fluorescence image stained FDA/PI; and (**B**) cell viability for 3, 5, and 7 days.

Cytotoxicity to the CHA-reinforced scaffolds was evaluated using a cell live/dead assay 7 days after cell seeding on the scaffolds. At 7 days after cell seeding, the contents of the CHA and HA did not affect cytotoxicity and cell distribution. In addition, on the 7th day, the presence of an MC coating on the CHA-reinforced scaffold did not affect the cell distribution. However, the detection amount of PI in the MC-coated scaffolds was lower than that of the MC-uncoated scaffolds (Figure 4A). As a result of the cell viability for 3, 5, and 7 days, it was possible to confirm a larger number of living cells in the MC-coated scaffold group.

### 2.7. Alkaline Phosphatase Activity of the CHA-Reinforced Scaffolds 

**Figure 5:**
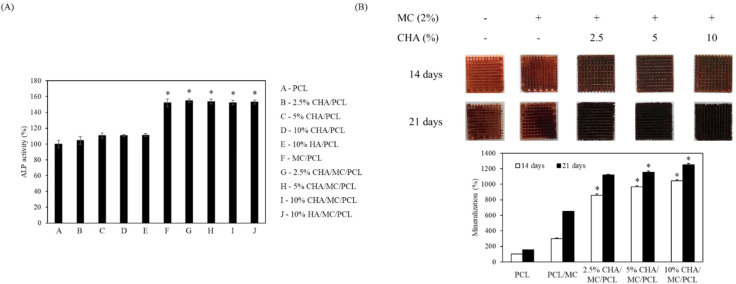
In vitro effect of scaffolds on (**A**) ALP activity and (**B**) mineralization activity during osteogenic differentiation of MC3T3-E1 cells. * *p* < 0.05 was considered to indicate a statistically significant difference compared with non-coated scaffolds.

The ALP activities of PCL, 2.5% CHA/PCL, 5% CHA/PCL, 10% CHA/PCL, 10% HA/PCL, MC/PCL, 2.5% CHA/MC/PCL, 5% CHA/MC/PCL, 10% CHA/MC/PCL, and 10% HA/MC/PCL scaffolds were 100 ± 5.08, 104.63 ± 4.91, 110.78 ± 3.28, 100.59 ± 1.27, 111.31 ± 1.81, 152.20 ± 5.09, 154.74 ± 2.59, 153.75 ± 3.26, 152.50 ± 2.62, and 153.05 ± 2.38, respectively. These results confirm that the ALP activity was increased in the MC-coated group on the scaffold (Figure 5A).

### 2.8. Mineralization of the CHA-Reinforced Scaffolds 

The PCL, MC/PCL, 2.5% CHA/MC/PCL, 5% CHA/MC/PCL, and 10% CHA/MC/PCL scaffolds were stained with Alizarin Red S stain, and calcium deposition on the scaffolds was observed. Along with the increasing concentration of CHA, the scaffolds created calcium in a dose-dependent manner. At 21 days, the 10% CHA/MC/PCL scaffold showed eight times more mineralization than the PCL scaffold (Figure 5B).

### 2.9. In Vivo Experiments

To confirm the bone regeneration ability of the scaffolds, the PCL, 10% CHA/MC/PCL, and 10% HA/MC/PCL scaffolds were implanted into a defect in the mouse calvarial defect model. 

**Figure 6:**
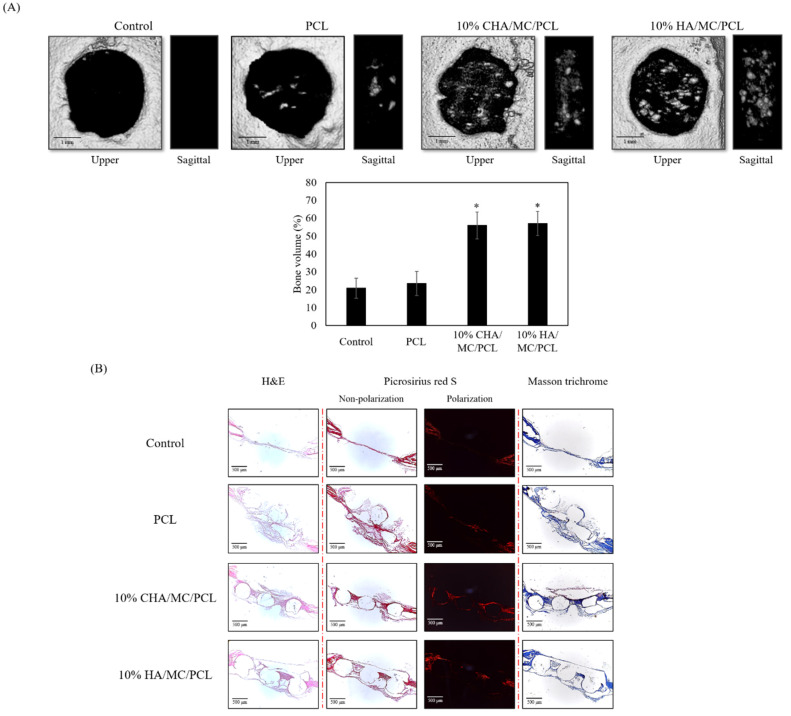
In vivo performance evaluation. **(A)** 3D reconstruction images of bone defect areas and (**B**) histological analysis of the effect of scaffolds on bone regeneration in vivo. Note: “S” represents scaffold; “black arrow” indicates new bone; “white arrow” indicates new type I collage. * *p* < 0.05 was considered to indicate a statistically significant difference compared with PCL group.

In the defect site, a difference in bone regeneration was confirmed by micro-CT between the non-treatment, PCL, CHA, and HA reinforced scaffolds. Twenty weeks after surgery, the area of the bone defect was determined by micro-CT. The 10% CHA/MC/PCL and 10% HA/MC/PCL scaffolds showed better regeneration, due to the synergistic effects of their 3D structure with CHA and MC for promoting bone regeneration, than the non-treatment group. Further investigation of the 3D reconstruction was conducted by analyzing the bone defect areas. This analysis showed that the bone volume of the 10% CHA/MC/PCL and 10% HA/MC/PCL scaffold groups have a greater quantity of bone volume regenerated than those of the non-treatment group (Figure 6A).

Histological analysis was performed with hematoxylin and eosin (HE), picrosirius red, and Masson’s trichrome (MT) staining of the bone defect area. At low magnification, the new bone and host bone were separated to define a 3-mm bone defect. Although the bone defect did not completely regenerate, the CHA- and HA-reinforced scaffold groups showed better regeneration than the non-treatment and PCL scaffold groups. As shown in the HE and MT staining images (Figure 6B), both CHA- and HA-reinforced scaffold groups formed regenerative tissue around the scaffolds. In addition, picrosirius red staining confirmed that the collagen formation around the scaffold was improved.

## 3. Discussion

The increasing global demand for fish has caused an increase in fish byproducts, such as fish scales, bones, and skins. Studying these byproducts as biomaterials can reduce the number of fish byproducts and increase their value [33]. Although not conducted in this study, it is necessary to study the selection of materials suitable for tissue engineering by comparing the characteristics of the collagen derived from terrestrial organisms and the collagen derived from marine organisms. In the present study, we isolated and characterized MC and CHA from *P. olivaceus* using pepsin hydrolysis and alkaline hydrolysis, respectively [34]. Numerous studies have reported about HA and collagen extracted and isolated from different fish species, such as *Oncorhynchus keta*, *Thunnus obesus,* and *Oreochromis* [35,36,37]. Unlike thermally extracted HA, HA extracted through alkaline hydrolysis retains its carbonate groups, because carbonate cannot be separated without heat treatment [36]. Furthermore, CHA was reported to have a similar chemical composition to that of HA present in natural bone tissue [38]. According to previous studies, the HA derived from fish bones shows improved biocompatibility and osteogenic differentiation activity, and enhanced potential for bone formation on scaffolds fabricated with HA. Thus, CHA is a potential biomaterial that can be employed with various synthetic or natural polymers to fabricate tissue-engineered bone substitutes for bone tissue regeneration applications [39]. 

XRD, FTIR spectroscopy, and EDS analyses of isolated CHA and commercially available HA were performed to compare and determine the crystallinity, chemical composition, and atomic percentage, respectively. The XRD analysis of CHA showed the same peak international standard as HA (ICDD 01-086-0740) at 002, 211, 112, 202, 310, 222, 213, 321, and 004 of Bragg’s reflection in the standard [35]. In addition, the results of both FTIR spectroscopy and EDS clearly indicated that the isolated CHA has a carbonate group attached to the HA unit, as shown in Figure 2A and Figure 3. In Figure 2A, a larger band value of CHA was confirmed at 1400–1500 cm^−1^, which appears to be because the carbonate group increased [40]. The relatively large value of these two bands (1400–1500 cm^−1^) is because CHA has a relatively higher carbonate group than HA. EDS analysis was performed to evaluate the presence of trace elements, such as Ca and P, belonging to the isolated CHA and commercial HA, and the results showed a Ca/P weight ratio of 1.96 and1.578 for CHA and HA, respectively (Figure 3), indicating that the extraction process (alkaline hydrolysis) of CHA has affected the Ca/P ratio. However, according to previous studies, the Ca/P ratios of both CHA and HA are not significantly different, and they were within the accepted range for hydroxyapatite [39].

Collagen was extensively used to fabricate various tissue regenerative substitutes, because it is one of the main components of the ECM and is an excellent biomaterial that provides exceptional biological and functional properties, without an associated inflammatory response or cytotoxicity [21]. In particular, MC is an alternative and attractive type of collagen over land animal-derived collagen for tissue engineering applications, since it does not have religious restrictions and is not associated with a risk of disease transmission to humans. Hence, we extracted MC from *P. olivaceus* and characterized it using UV/Vis spectra, SDS-PAGE, FTIR spectroscopy, and amino acid composition analysis. The UV/Vis spectra recorded a relatively low absorption value at approximately 280 nm compared with general proteins, because collagen contains a relatively lesser amount of tyrosine and phenylalanine than general proteins (Figure 1A). Moreover, the SDS-PAGE results showed two distinguishable bands at approximately 130 kDa, corresponding to the α1 and α2 chains, and two bands at 250 kDa and 310 kDa, corresponding to the larger β and γ chains, respectively, suggesting that extracted *P. olivaceus* skin collagen is type I collagen, which is in agreement with the findings of previous studies [21]. The FTIR spectra of the extracted MC showed five characteristic peaks at 3300 cm^−1^, 2972 cm^−1^, 1635 cm^−1^, 1549 cm^−1^, and 1239 cm^−1^, which correspond to amide A, amide B, amide I, amide II, and amide III, respectively, similar to that of commercial collagen and the findings of previous publications [41]. Type I collagen forms a triple-helix structure with 20 different amino acids and is stabilized by its high content of glycine repeated every three residues, proline, and hydroxyproline. Moreover, proline and hydroxyproline showed lower values in MC, which is in agreement with the findings of previous reports [42]. Overall, the results suggested that MC and CHA were successfully extracted and characterized from *P. olivaceus*.

According to previous reports, various synthetic and natural biocompatible materials were employed to fabricate 3D-printed scaffolds and demonstrated good bone tissue regeneration effects. Among them, many researchers have focused on improving biological activities including cell adhesion, proliferation, migration, and differentiation through surface modifications including surface coating with natural or chemically modified biocompatible materials [43]. In the present study, we fabricated a 3D-printed porous PCL scaffold reinforced with CHA and surface-coated with MC to enhance osteogenic differentiation. To evaluate the osteogenic activity of the fabricated scaffolds, MC3T3-E1 cell-seeded scaffolds were analyzed, using ALP assay and Alizarin Red S staining. According to the results, the CHA/MC/PCL scaffolds significantly enhanced the mineral deposition through differentiation of MC3T3-E1 pre-osteoblasts to osteoblasts compared with the pure PCL scaffold, indicating that MC and CHA have excellent osteogenic activities and excellent synergetic effects on bone tissue regeneration. Moreover, the bone tissue regeneration effects of the fabricated scaffolds were evaluated using an in vivo calvarial defect mouse model. Micro-CT and histological analysis indicated that the 10% CHA/MC/PCL- and 10% HA/MC/PCL-treated groups had prominent bone tissue reconstruction effects, compared with the non-treatment and PCL-treated groups (Figure 6). Overall, these results suggest that materials obtained from marine byproducts have considerable potential for bone tissue regeneration and are attractive alternatives for terrestrial or synthetic materials. Based on this study, the possibility was evaluated of MC- and CHA-derived byproducts from *P. olivaceus* used as a substitute material for bone tissue. Furthermore, we will proceed with research on bone mimic scaffold containing MC and CHA. 4. 

## 4. Materials and Methods

### 4.1. Materials

The by-product from *P. olivaceus* was provided by EUNHA Marine Co., Ltd. (Busan, Korea). The α-minimum Eagle’s medium (α-MEM), fetal bovine serum (FBS), trypsin (250 U/mg), penicillin/streptomycin, and other materials used in cell culture experiments were purchased from GIBCO™ (Gaithersburg, MD, USA). Polycaprolactone (PCL), 1-Step p-nitrophenyl phosphate (pNPP), and Alizarin Red S were purchased from Sigma-Aldrich (St. Louis, MO, USA). The other chemical reagents and materials that were used were commercially available and analytical grade.

### 4.2. Extraction and Characterization of Pepsin Soluble MC 

#### 4.2.1. Extraction of Pepsin Soluble MC

**Figure 7:**
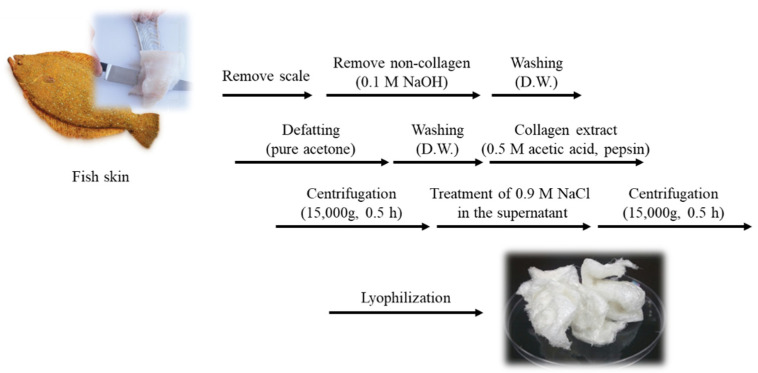
MC extraction methods from *P. olivaceus* (pepsin hydrolysis).

*P. olivaceus* skin was descaled and desalted by washing with cold water at 4 °C for one day and cut into small pieces. Pepsin-soluble MC was extracted from the prepared skin, following the method described by [44] with slight modifications. All steps of the procedure were carried out at 4 °C with gentle stirring. Non-collagenous proteins were removed with 0.1 M NaOH at small pieces to a solution ratio of 1:10 (*w/v*) for 2 days. The skins were then washed with ultrapure water until they became a neutral pH. The skins were defatted with acetone with a pieces to solution ratio of 1:10 (*w/v*) for 2 days with a changing to new acetone solution every 12 h, and then thoroughly washed with ultrapure water. Then the skins were suspended in 0.5 M acetic acid with a pieces to solution ratio of 1:20 (*w/v*) for 1 day. After pretreatment, the fish skin was hydrolyzed by pepsin to extract collagen. The skin was dissolved in 0.5 M acetic acid with pepsin (pepsin 1:3000, Sigma, St. Louis, MO, USA) for 24 h at 4 °C, then centrifuged at 15,000 rpm for 30 min. The supernatant was salted out by adding NaCl until a final concentration of 0.9 M. The resultant precipitate was collected by centrifugation at 15,000 rpm for 1 h and then dissolved in 0.5 M acetic acid. The solution was then dialyzed against 0.1 M acetic acid for 1 day and ultrapure water for 3 days. The resultant dialysate was lyophilized and was referred to as MC (Figure 7).

#### 4.2.2. Sodium Dodecyl Sulfate Polyacrylamide-Gel Electrophoresis (SDS-PAGE)

SDS-PAGE was performed, following the method of [45] with slight modifications, using 7.5% separating and 5% stacking gel. The collagen samples were dissolved in the sample buffer and the obtained mixture (1 mg/mL) was heated at 100 °C for 5 min. The mixture was centrifuged at 4000 rpm for 5 min using a microcentrifuge at room temperature to remove debris. A total of 20 μg of the sample was loaded onto a polyacrylamide gel and subjected to electrophoresis at a constant voltage (100 V) for 1 h using MiniProtein II unit (Bio-Rad Laboratories, Inc. Richmond, CA, USA). The resultant gel was stained with 0.1% (*w/v*) Coomassie blue R-250 in 50% (*v/v*) methanol and 10% (*v/v*) acetic acid for 2 h and destained with 40% (*v/v*) methanol and 10% (*v/v*) acetic acid. High molecular weight markers were loaded alongside the collagen to estimate the molecular weight of MC, and commercial atelocollagen (Atelocollagen, Dalim Tissen, Korea) was loaded next to the protein marker as standard collagen.

#### 4.2.3. UV Absorbance Analysis

The UV absorption spectra of MC from the skin of *P. olivaceus* were studied, following the method reported elsewhere with slight modifications [46]. The MC and commercial atelocollagen samples (1 mg) were dissolved in 1 mL of 0.5 M acetic acid and the collagen solutions were centrifuged at 15,000 rpm for 10 min at 4 °C. The collagen solution was placed in a quartz cell with a path length of 1 mm. The collagen solutions were subjected to absorbance at wavelengths between 200 and 500 nm at a scan speed of 2 nm per second with an interval of 1 nm. All spectra were obtained using a UV–visible spectrometer (Epoch 2 Microplate reader, Biotek, Winooski, VT, USA).

#### 4.2.4. Amino Acid Contents

Amino acid compositions were analyzed using an automatic analyzer (Hitachi Model 835-50, Tokyo, Japan) with a C18 column (5 μm, 4.6 × 250 nm, Watchers, MA, USA). The reaction was carried out at 38 °C, with the detection wavelength at 254 nm and flow rate of 1.0 mL/min. All chemical analyses (from each tank) were carried out in triplicate.

### 4.3. Isolation and Characterization of Carbonated Hydroxyapatite

#### 4.3.1. Isolation of Carbonated Hydroxyapatite from *P. Olivaceus*

**Figure 8:**
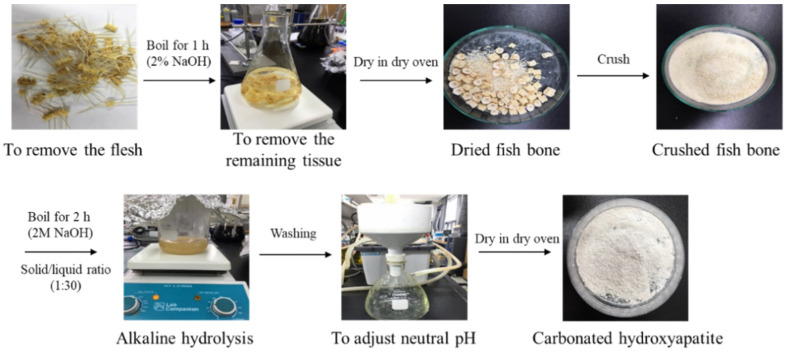
CHA extraction methods from *P. olivaceus* (Alkaline hydrolysis).

*P. olivaceus* bones were cut into small pieces using a bladed cutter. The bone pieces were boiled in 100 °C purified water for 1 h to remove unnecessary parts. Then, the bone pieces were boiled in 10 mL of acetone and 2% NaOH for 1 h, and the water was completely removed at 100 °C. The dried bone pieces were crushed using a homogenizer. CHA was extracted by boiling the crushed bone pieces for 1 h in 200 °C 2 M NaOH to completely remove the organic materials. Collected CHA was washed with purified water to adjust the pH to neutrality and to remove all of the moisture from the dry oven (Figure 8).

#### 4.3.2. Fourier Transform Infrared (FTIR) Spectroscopy

FTIR spectroscopy (Perkin Elmer, Waltham, MA, USA) data were collected from raw fishbones, CHA, HA, MC, and commercial atelocollagen to determine the functional groups of hydroxyapatite and collagen. The IR spectra represent the average of 30 scans between 500 cm^−1^ and 4000 cm^−1^, at a resolution of 4 cm^−1^.

#### 4.3.3. X-ray Diffraction (XRD) Analysis

XRD analysis was conducted on the fishbones, HA and CHA using an Ultima IV system (Rigaku Co., Tokyo, Japan) with Cu-Kα radiation. The X-ray diffraction intensities were recorded within the range of 5 to 80°, at a scanning rate of 2° min^−1^. 

### 4.4. Fabrication and Characterization of 3D Scaffolds

**Figure 9:**
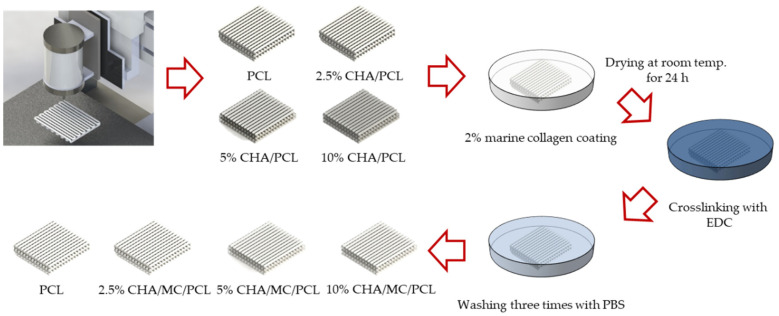
Schematic diagram of CHA-reinforced PCL scaffold and coated collagen.

In this study, we used a computer-controlled three-axis robot system (EZ-ROBO-5GX ST2520, Iwashita Engineering Inc., Fukuoka, Japan), supplemented with a dispenser to fabricate the PCL, HA/PCL, and CHA/PCL structure. The PCL struts were melted at 100 °C in a heating barrel and were extruded through a heated 21G nozzle at a constant pressure (500 ± 25 kPa). Following this condition, the PCL struts were built in a layer-by-layer manner to make a 3D structure with uniform height and porosity. After fabricating the multilayered structure, the fabricated scaffold was sterilized in 70% EtOH. After fabricating the PCL, the HA/PCL and CHA/PCL scaffolds were coated with MC on the surface through 1-ethyl-(3-3-dimethylaminopropyl) carbodiimide hydrochloride (EDC) coupling reaction (Figure 9).

#### 4.4.1. Scanning Electron Microscope (SEM) Analysis

The structural morphologies of PCL, 2.5% CHA/PCL, 5% CHA/PCL, 10% CHA/PCL, 10% HA/PCL, MC/PCL, 2.5% CHA/MC/PCL, 5% CHA/MC/PCL, 10% CHA/MC/PCL, and 10% HA/MC/PCL scaffolds were examined using a field emission scanning electron microscope (SEM, Tescan, Czech, VEGA II LSU) at 15 kV. The diameter of the 3D scaffolds was measured from the SEM image using image analysis software (Image J, National Institutes of Health, Bethesda, MD, USA).

#### 4.4.2. Tensile Properties

The tensile properties of PCL, 2.5% CHA/PCL, 5% CHA/PCL, 10% CHA/PCL, 10% HA/PCL, MC/PCL, 2.5% CHA/MC/PCL, 5% CHA/MC/PCL, 10% CHA/MC/PCL, and 10% HA/MC/PCL scaffold (2.5 × 2.5 mm^2^) were measured using a universal tensile machine (Top-tech 2000, Chemilab, Kimpo, Korea). The PCL scaffolds were mounted and subjected to a crosshead speed of 0.2 mm/s at room temperature until failure. The elastic modulus was investigated by the average of three runs for each scaffold.

### 4.5. In Vitro Study on Fabricated Scaffolds

#### 4.5.1. Cell Culture and Cell Viability

The MC3T3-E1 subclone 4 cells were purchased from the American Type of Culture Collection (Rockville, MD, USA). The MC3T3-E1 cells were cultured in α-MEM without ascorbic acid, supplemented with 10% fetal bovine serum (FBS), 100 μg/mL streptomycin, and 100 U/mL penicillin. The MC3T3-E1 cells were incubated in 5% CO_2_ humidified atmosphere and at 37 °C and sub-cultured every 2–3 days.

The MC3T3-E1 cells were seeded onto the scaffolds by dropping them onto scaffolds at a density of 1 × 10^5^ per scaffold. Before the cells were seeded, the scaffolds were sterilized with 70% ethyl alcohol (EtOH) and UV light.

The cell viability and distribution of the MC3T3-E1 pre-osteoblasts cultured on the CHA-reinforced scaffolds were determined by Cell Counting Kit-8 (CCK-8) assay (Dojindo, Kumamoto, Japan) and live/dead fluorescence staining. Cell viability at 3, 5, and 7 days via CCK-8 assay was evaluated, according to the manufacturer’s protocol. A total of 10 μL of CCK-8 solution along with 100 μL of α-MEM were added to each well; the mixture was incubated at 37 °C. for 2 h. The cell viability was evaluated at 450 nm by using a microplate reader (Biotek, Winooski, VT, USA). The cell viability was evaluated using a fluorescence microscope through the Live/Dead assay. After 7 days, the MC3T3-E1 seeded on the CHA-reinforced scaffolds were stained with fluorescein diacetate (8 μg/mL) and propidium iodide (20 μg/mL) for 15 min at room temperature after washing three times with PBS. The stained MC3T3-E1 on the scaffolds were qualitatively examined under a fluorescence microscope (Axio Observer A1, Zeiss, Jena, Germany).

#### 4.5.2. ALP Activity and Mineralization Analysis on 3D Scaffolds

MC3T3-E1 cells were cultured in α-MEM containing 50 μg/mL ascorbic acid and 10 mM β-glycerophosphate for the osteogenic differentiation. For the incubation, the osteogenic differentiation media was changed every 2 days. 

After 7 days, the 1-StepTM PNPP reagent (100 μL) was added to wells and incubated for 30 min. The 2N NaOH solution was added to stop reactions. The absorbance was measured at 405 nm with a microplate reader. Mineralization in the scaffolds was calculated by subtracting the Alizarin Red values, which were stained by the HA and CHA contained in the cell-free scaffolds.

After 14 and 21 days, the MC3T3-E1 cells were fixed with 10% formalin and stained with Alizarin Red S (40 mM). After staining with Alizarin Red S, the Alizarin Red S was removed and washed three times with D.W. The staining was dissolved through cetylpyridinium chloride and the absorbance of the dissolved stain was measured at 550 nm using a microplate reader.

### 4.6. In Vivo Study in Mouse Calvarial Defect Model

The experimental protocol was approved by the Animal Care and Experiment Committee of Pukyong National University and performed following relevant guidelines and regulations for the care and use of laboratory animals. To determine the bone regeneration ability of the containing MHA and MC, Male CrljOri:CD1 (ICR) mice (approximate weight of 35 g) were used as an in vivo bone defect model. The experiment was conducted according to the protocols approved by the Institutional Animal Care and Use Committee. The ICR mice were maintained on a 12 h light/dark cycle in a controlled environment (relative humidity: 40–70%; temperature: 20–24 °C).

#### 4.6.1. Mouse Calvarial Defect Model and Implantation of 3D Scaffolds

Anesthesia of ICR mice was performed by intraperitoneal injection of Zoletil 50. After brightening and disinfecting the upper part of the head, the surgical site was vertically incised and the soft tissue was removed to expose the calvarium. Two 3 mm diameter defects created on both side in the exposed calvarium and PCL, 10% HA/MC/PCL, and 10% CHA/MC/PCL scaffolds were implanted into the bone defect site. The non-treated group was used as a control group. 

#### 4.6.2. Micro-Computed Tomography Analysis

After the calvarial defect was created, it was implanted with PCL, 10% HA/MC/PCL, and 10% CHA/MC/PCL scaffolds. After 20 weeks of operation, ICR mice were euthanatized and the calvarial specimens were harvested and fixed in 10% formalin for further characterization and analysis. Micro-CT (NFR Polaris-G90, NanoFocusRay Co., Ltd., Korea) was firstly used to detect the defect area with the settings (80 kV, 0.06 mA). The 3D structures of calvarium were reconstructed through Mimic software (Radiant, Poznan, Poland).

#### 4.6.3. Histological Analysis

Calvarial specimens were fixed in 10% formalin for 5 days at room temperature and decalcified in 8% formic acid and 8% hydrochloric acid, frozen in mounting media. The frozen blocks were cut into 5 μm-thick sections across the center of the defect area and stained with HE, picrosirius red, and MT staining to evaluate the bone regeneration.

### 4.7. Statistical Analysis

All quantitative data are presented as means ± standard deviation (SD) with at least three individual experiments that were conducted using fresh reagents. Significant differences among the groups were determined using the unpaired Student’s *t*-test. The differences were considered statistically significant at *p* < 0.05.

## 5. Conclusions

In this study, MC and CHA were extracted from *P. olivaceus* byproducts (skin and bone), and a 3D scaffold reinforced with CHA and coated with MC was fabricated to evaluate bone regeneration. First, CHA and MC extracted from the *P. olivaceus* byproducts were analyzed to determine that they had the characteristics of HA and collagen. By fabricating a 3D scaffold, osteogenic differentiation was confirmed using MC3T3-E1 cells, and bone regeneration was confirmed in a mouse calvarial defect model. Micro-CT and histological analysis revealed that the tissue regeneration of the defect site in the 10% HA/MC/PCL and 10% CHA/MC/PCL groups was superior to that in the non-treatment and PCL scaffold groups. These results suggest that marine byproduct-derived materials can be valuable alternatives for land animal-derived materials. Based on the results, further study is needed to develop bone mimic scaffold with blood vessels, in order to understand bone regenerative mechanism.

## Figures and Tables

**Table 1:** Amino acid composition of atelocollagen obtained from the skins of *P. olivaceus* and of porcine commercial atelocollagen (per 1000 residues).

Amino Acid	Porcine Atelocollagen	Marine Atelocollagen
Asp	53.6	56.2
Thr	17.9	27.4
Ser	33.1	44.8
Glu	96.0	95.6
Gly	238.3	247.3
Ala	91.0	108.2
Cys	1.6	2.0
Val	18.3	16.6
Met	5.7	11.0
Iie	9.4	6.9
Leu	28.8	23.7
Tyr	1.2	2.1
Phe	18.4	20.5
Lys	35.9	36.9
His	6.4	7.3
Arg	80.0	84.8
Hypro	126.0	96.9
Pro	138.3	111.7
Total	1000.0	1000.0

**Table 2:** Strut diameter and elastic modulus of CHA-reinforced scaffolds.

Scaffolds	Strut Diameter (μm)	Elastic Modulus (MPa)
PCL	518.54 ± 5.72	6.29 ± 0.28
2.5% CHA/PCL	607.27 ± 3.34	10.19 ± 0.01
5% CHA/PCL	571.37 ± 16.43	9.26 ± 0.33
10% CHA/PCL	561.03 ± 16.5	6.85 ± 0.55
10% HA/PCL	484.57 ± 19.68	7.76 ± 0.37
MC/PCL	508.17 ± 11.62	6.37 ± 0.16
2.5% CHA/MC/PCL	599.42 ± 2.94	9.38 ± 0.45
5% CHA/MC/PCL	526.28 ± 15.34	9.1 ± 0.12
10% CHA/MC/PCL	539.12 ± 15.34	7.08 ± 0.52
10% HA/MC/PCL	466.67 ± 16.04	7.77 ± 0.42

## Data Availability

Not applicable.
